# Restricted Application of Insecticides: A Promising Tsetse Control Technique, but What Do the Farmers Think of It?

**DOI:** 10.1371/journal.pntd.0001276

**Published:** 2011-08-09

**Authors:** Fanny Bouyer, Seyni Hamadou, Hassane Adakal, Renaud Lancelot, Frédéric Stachurski, Adrien M. G. Belem, Jérémy Bouyer

**Affiliations:** 1 Centre International de Recherche-Développement sur l'Elevage en zone Sub-humide (CIRDES), Bobo-Dioulasso, Burkina Faso; 2 Direction de l'Agriculture et de la Sécurité Alimentaire (DASA), Département Développement Rural, Ressources Naturelles et Environnement (DDRE), Union Economique et Monétaire Ouest Africaine (UEMOA), Ouagadougou, Burkina Faso; 3 UMR Contrôles des Maladies Animales et Émergentes, Centre de Coopération Internationale en Recherche Agronomique pour le Développement (CIRAD), Campus International de Baillarguet, Montpellier, France; 4 Institut du Développement Rural (IDR), Université Polytechnique de Bobo-Dioulasso (UPB), Bobo-Dioulasso, Burkina Faso; 5 Institut Sénégalais de Recherches Agricoles, Laboratoire National d'Elevage et de Recherches Vétérinaires, Hann, Dakar, Sénégal; Foundation for Innovative New Diagnostics (FIND), Switzerland

## Abstract

**Background:**

Restricted application of insecticides to cattle is a cheap and safe farmer-based method to control tsetse. In Western Africa, it is applied using a footbath, mainly to control nagana and the tick *Amblyomma variegatum*. In Eastern and Southern Africa, it might help controlling the human disease, i.e., Rhodesian sleeping sickness as well. The efficiency of this new control method against ticks, tsetse and trypanosomoses has been demonstrated earlier. The invention, co-built by researchers and farmers ten years ago, became an innovation in Burkina Faso through its diffusion by two development projects.

**Methodology/Principal Findings:**

In this research, we studied the process and level of adoption in 72 farmers inhabiting the peri-urban areas of Ouagadougou and Bobo-Dioulasso. Variables describing the livestock farming system, the implementation and perception of the method and the knowledge of the epidemiological system were used to discriminate three clusters of cattle farmers that were then compared using indicators of adoption. The first cluster corresponded to modern farmers who adopted the technique very well. The more traditional farmers were discriminated into two clusters, one of which showed a good adoption rate, whereas the second failed to adopt the method. The economic benefit and the farmers' knowledge of the epidemiological system appeared to have a low impact on the early adoption process whereas some modern practices, as well as social factors appeared critical. The quality of technical support provided to the farmers had also a great influence. Cattle farmers' innovation-risk appraisal was analyzed using Rogers' adoption criteria which highlighted individual variations in risk perceptions and benefits, as well as the prominent role of the socio-technical network of cattle farmers.

**Conclusions/Significance:**

Results are discussed to highlight the factors that should be taken into consideration, to move discoveries from bench to field for an improved control of trypanosomoses vectors.

## Author Summary

Restricted application of insecticides to cattle is a cheap and safe farmer-based method to control tsetse and the diseases they transmit, i.e. human and animal African trypanosomoses. The efficiency of this new control method has been demonstrated earlier but no data is available on its perception and adoption intensity by farmers. We studied these two features in Burkina Faso, where the method has diffused thanks to two development projects. The study allowed identifying three groups of farmers with various adoption intensities, of which one was modern and two traditional. The economic benefit and the farmers' knowledge of the epidemiological system appeared to have a low impact on the early adoption process whereas some modern practices, as well as social factors appeared critical. The quality of technical support provided to the farmers had also a great influence on the adoption rate. The study highlighted individual variations in risk perceptions and benefits, as well as the prominent role of the socio-technical network of cattle farmers. The results of the study are discussed to highlight the factors that should be taken into consideration, to move discoveries from bench to field for an improved control of trypanosomoses vectors.

## Introduction

Tsetse flies (Diptera: Glossinidae) are the vectors of human and animal African trypanosomoses, the former a major neglected disease, and the latter considered among the greatest constraints to livestock production in sub-Saharan Africa. The integrated management of these diseases would require the combination of tsetse control with trypanocide treatments. In 2001, an African Union initiative called the Pan African Tsetse and Trypanosomosis Eradication Campaign (PATTEC) was launched following an historic decision by the African Heads of State and Government in Lome, Togo, July 2000 (http://www.africa-union.org/Structure_of_the_Commission/depPattec.htm). Various national initiatives joined this campaign, including in Burkina Faso, where the Government has embarked on an ambitious tsetse eradication campaign that targets the northern Mouhoun River Basin for its first phase (http://www.pattec.bf/index1.php). Considering the large areas infested by tsetse, this goal will however require the sustainable involvement of final beneficiaries, i.e. farmers. A number of efficient tsetse control tactics are available, but unfortunately none are widely used by farmer communities. The gap between solutions and research discoveries on the one hand, and changes in farming practices on the other hand is generally huge in the field of agriculture in Africa, and particularly so regarding the control of tsetse and African trypanosomoses [1]. Research-built solutions, i.e. «technological recipes» that may be very efficient in experimental conditions, are often not adopted by farmers: invention does not necessarily lead to innovation [2]. There is thus still room for innovation and a need to understand the factors favouring or hampering the innovation process. During the recent years, two major inventions were proposed within the field of tsetse control: the use of mosquito netting impregnated with pyrethroids and placed around cattle or pig pens [3] and the restricted application of insecticides to cattle extremities [4]–[6]. While insecticide fences have recently contributed to the reduction of tsetse populations by 100% by a national program targeting Loos Islands in Guinea [7], restricted application of insecticides has been recognized as a cheap, safe and environment friend farmer-based method to control tsetse and trypanosomoses in general [4], [8], and Rhodesian sleeping sickness in particular [9]. In Burkina Faso, this method is applied using footbaths that allow treating large herds within a short time and has been diffused by two development projects (see below).

### Tsetse and trypanosomoses in Burkina Faso

Human sleeping sickness has almost disappeared from Burkina Faso, thanks to the sterilization of the parasite reservoir through medical surveys during the colonization and just after the independence. The combination of environmental and predominantly demographic factors then allowed to keep this result by reducing tsetse distribution and abundance and the contact between human and tsetse [10]. Tsetse however remain present in a large part of the country [11], representing a permanent risk of re-emergence of the disease thanks the immigration of infected persons from endemic countries, particularly Ivory Coast, where social conflicts favors emigration especially towards Burkina Faso (non autochthonous cases are reported every year) [12]. Moreover, animal trypanosomoses (Nagana) represent heavy economic burdens for the farmers and the national economy. Livestock farming is actually the main or secondary occupation for 86% of the population in Burkina Faso. It generates 12% of the Gross Domestic Product (GDP) and 19% of the export income [13]. Moreover, animal traction is also widely used for crop cultivation of cotton and cereals which provide 40% of the GDP.

Nagana is identified by the farmers as the main health constraint to cattle farming in south-western Burkina Faso [14]. Its control is based on the use of curative or preventive trypanocides, leading to an increased risk of chemoresistance. Farmers' knowledge of the vectors is poor, and tsetse control is considered by the population as a public good. Generally, a vector control technique that is not using individual animal treatments is not adopted by the farmers [15].

### History of footbaths in Burkina Faso

Originally, restricted application of insecticides using a footbath was designed to control *Amblyomma variegatum* (Acari: Ixodidae) at the International centre for livestock research and development in sub-humid areas (CIRDES), based at Bobo-Dioulasso in Burkina Faso. Actually, *Amblyomma variegatum* is the most harmful hard-tick species for ruminants, causing direct losses [16], transmitting *Ehrlichia ruminantium* - the causative agent of heartwater, and favoring the clinical expression of dermatophilosis caused by *Dermatophilus congolensis*. Farmers are aware of cattle losses caused by this tick. They use individual control methods such as manual removal (time consuming), insecticide spraying and pour-on application (both expensive). Because footbaths do not eliminate all the attached ticks, there is no risk to break the enzootic stability of cowdriosis.

Behavioral ecology studies have revealed that *A. variegatum* first attach to the inter-digital areas of cattle legs before reaching its preferred attachment sites – udder and lower part of the abdomen, and the perineal region, when cattle lie down to rest. This observation was at the origin of the use of restricted application of insecticides to cattle using a footbath [17], [18].

Thereafter, footbaths also proved efficient against tsetse that present a tropism towards the distal parts of cattle legs [6], [19]. For instance, repeated and restricted pyrethroid-based footbath treatments allowed reducing nagana incidence by 90% in a peri-urban area of Burkina Faso [19]. However, this method is based on strict technical recommendations, and it is a prophylactic and individual control method against ticks [17], and a collective one against tsetse flies. As a matter of fact, it is necessary to treat a large proportion of cattle in a given area to effectively reduce tsetse population [19]–[21].

To assess whether this method could be transferred to targeted farmers, two experimental footbaths were built in villages close to CIRDES, during a participatory approach with two groups of farmers called “action research” [22]. Transfer risks were mitigated, with financial and technical support provided to the farmers by the research center. A follow-up was implemented during 4 years, thus allowing the enhancement of the footbath by improving its design and accessories. The technical package resulting from interactions between scientists and farmers was published in papers targeting the farmers and presented in workshops to favor its diffusion [23], [24]. At first, this innovation was exogenous, but it can then be considered rather of mixed nature [25].

This process was pursued by two local livestock development projects. Their main objective was to strengthen the technical and economical capacities of the groups of livestock keepers (GLK). Following the analysis of their needs, the implementation of animal health services based on acaricide/insecticide footbaths was identified as a relevant action for improving cattle productivity and the whole production systems through the strengthening of GLK capacities. The actions promoting the diffusion of footbaths included workshops with GLK-elected members, field visits, hosting of GLK meetings, and strengthening between-farmers communication. The socio-technical network was thus reinforced to facilitate the implementation of footbaths that would in turn strengthen the GLK by creating a new service to their members. Financial, technical, and organizational guidelines were provided, including written specifications and training of the control committees (Bouyer F., pers. com.).

The development projects provided technical guidance and funding for the building of the footbaths which cost about euro 535 each (350.000 FCFA). The farmers paid 15% of this amount (collective or individual contributions) and provided labour, sand, water and local materials (wood) for the waiting pen. Each group of livestock keepers (GLK) created committees for maintenance and financial management of the footbaths that included two technical managers of the footbath trained at CIRDES and the treasurer of the GLK. In addition, a first liter of active ingredient (alpha-cypermethrin, Dominex, FMC, Philadelphia, USA) was provided and used for treatment at the recommended concentration (0,005%) [6]. The farmers then paid a treatment fee per head of cattle (5 to 10 FCFA i.e. euro 0.08 to 0.16) including the salary of the two managers of the footbath, the consumption of insecticide per head which was evaluated a posteriori using the treatment spreadsheets and a provision for depreciation of the footbath.

This study aimed at quantifying the footbaths adoption rates and factors in Burkina Faso to improve the future adoption of this new tsetse control method in the framework of the PATTEC initiative.

## Methods

### Study area and production systems

The study was carried out in Burkina Faso in the peri-urban areas of Ouagadougou (the capital city) and Bobo-Dioulasso (the second city), with a north sudanese climate for the former, and a south sudanese climate for the latter (700 and 1050 mm of mean annual rainfall respectively) [11]. *Amblyomma variegatum* was present in both areas [16].

Ouagadougou and Bobo-Dioulasso are located in the Kadiogo and Houet provinces respectively, where the cattle densities are 45 and 56 heads per square km. The human populations reach 543 and 78 inhabitants per square km respectively. The main ethnic groups are the Mossi in the area of Ouagadougou, and the Bwaba, Ko and Bobo in the area of Bobo-Dioulasso.

In the peri-urban area of Bobo-Dioulasso, trypanosomoses risk is considered as high, with a mean annual incidence of 76% in the absence of treatment [20]. On the contrary, the risk of nagana was almost null for the sedentary cattle farms of the peri-urban area of Ouagadougou, which could thus be considered as a negative control to measure the adoption rate of footbaths in the absence of tsetse. Actually, the latter disappeared in this area following a decrease of annual rainfall and degradation of their natural habitats [11].

Modern farms were mostly located in the peri-urban of Ouagadougou, in relation to lower health constraints and the proximity of a bigger market. Exotic cattle breeds were used in the farms belonging to the local dairy farmer association (Association des Promoteurs de Lait Local, APLL). Brazilian, European, and crossbred cattle with local zebus were found in these farms. Forage production or distribution was frequent, together with modern housing and farming facilities. Most of the interviewed farmers were Mossi in Ouagadougou (73%) but one was Fulani, one Gourmantché and one Songhaï. The mean herd size was 71 (s.d. 80).

In contrast, this production system was almost absent in the peri-urban area of Bobo-Dioulasso (<1%). Transhumant farmers using local zebus and few inputs were the most common (92%) [26]. Some farmers had however entered into an intensification strategy.

Most of the interviewed farmers were Fulani in Bobo Dioulasso (84%) but 13% were Bobo and one was Dioula and one Mossi. The mean herd size was 64 (s.d. 42).

### Field surveys

**Figure 1:**
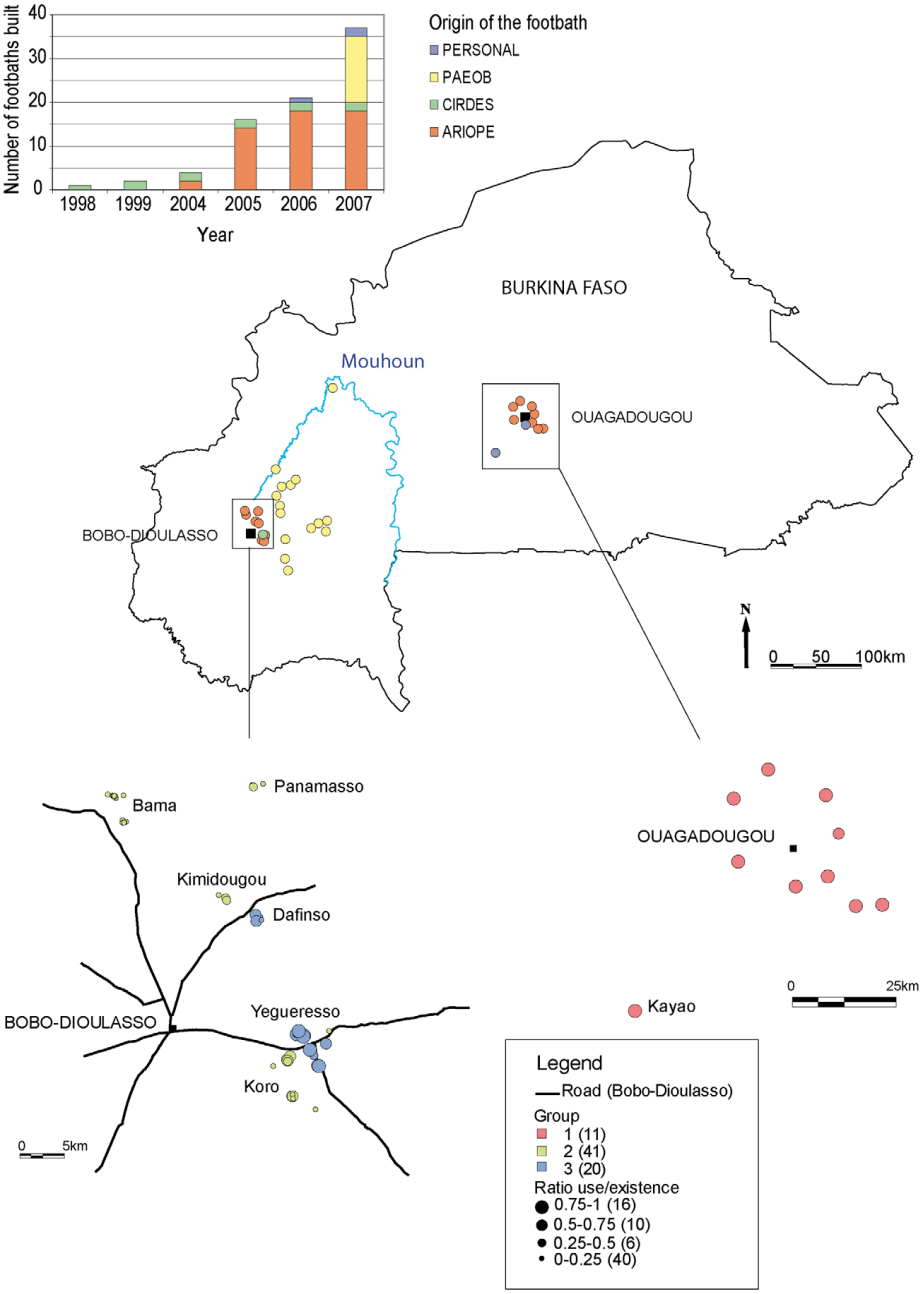
Location of the footbaths in Burkina Faso. At the top, footbaths are described according to the origin of their implementation: built by the Centre International de Recherche sur l'Elevage en zone Sub-humide (CIRDES) for research purposes, funded by the Projet d'Appui au renforcement institutionnel des organisations professionnelles d'éleveurs modernes (ARIOPE development project), funded by the Projet d'Appui à l'Elevage dans l'Ouest du Burkina (PAEOB development project), or built using personal funding. At the bottom, herds are located by their night pens, colored according to the farmer group, and sized according to the ratio of the duration of individual use on the duration of the footbath existence.

The survey was carried out in 2008 at the end of the dry season and the beginning of the rainy season. Only the footbaths built before 2007 were enrolled in the sample, since it was not appropriate to assess the adoption within the first year of installation. Footbaths that were not built or used to control vectors were also excluded. All footbaths were identified and georeferenced (Fig. 1). Potential users of a footbath were defined as:

1. the members of a GLK in which a footbath was built, and whose night cattle pens were located <2 km from the footbath,
2. Other farmers that used a footbath but were not GLK members,
3. In the case of individual footbaths, the owner only was involved in the survey.

All the members of the beneficiary GLK and approximately half of the non members were surveyed, totalizing 22 footbaths and 72 farmers.

Three kinds of questionnaires were used: one on “community life”, one on “technical and financial management of the footbath”, and one describing the farmer.

The “community life” questionnaire involved the elected people from each GLK where at least one individual or collective footbath was implemented. The questions asked were about the process of footbath implementation, the GLK organizational skills (kind of activities lead) and their vector control strategy (collective or not). A list of members was established and the footbaths and night pens located within 2 km around were georeferenced. The night pens of the debriefed non members were also georeferenced.

The “technical and financial management of the footbath” questionnaire was filled with at least one manager of the footbath or two elected people of the GLK for collective footbaths, and the owner for individual footbaths. Questions addressed the technical and financial management practices of the footbath. Footbath use was measured for the previous rainy season of use: number of herds and cattle treated, treatment frequency and annual duration of use. Quantitative data were retrieved from the footbath management documents (treatment spreadsheets).

The “farmer” questionnaire was filled during an individual interview with the person responsible for the herd (10% were herders and 90% the cattle owners). It included a farm typology, farmer's perception and knowledge of ticks, tsetse, and vector control strategies. Farmer's use and perception of the footbath, and quantitative data were also recorded: herd size, transhumance dates, and veterinary costs (for ticks and nagana control).

Farmers' knowledge of the epidemiological system was characterized with 11 qualitative variables describing the diagnosis of ticks and tsetse, the appraisal of their pathogenicity and vectorial importance, and the general knowledge of vectors (number of known vector species). These questions were derived from rapid African Animal Trypanosomosis (AAT) risk appraisal methods [27]. Dry-mounted insects and ticks (domestic flies, tsetse, tabanids, stomoxes, ticks *Amblyomma variegatum*, *Hyalomma sp.*, *Boophilus sp.*) were presented to the farmers in Petri dishes to evaluate their diagnosis skills.

### Statistical analyses

Variables with no or little variation were discarded from the statistical analysis, together with unreliable or incomplete data. Thus, 21 variables describing cattle farming practices and farmers'perceptions of footbaths (Table 1), and 11 variables describing farmers'knowledge of ticks and tsetse were kept for subsequent steps. These two sets of variables are thereafter called “cattle practices” and “knowledge of the epidemiological system”.

**Table 1:** Variables describing practices and perceptions of the farmers.

Category	Variables	Modalities
**Implementation of the service**	kind of waiting pen*	absence/stalling/round with wire netting/intermediate (funnel shaped with wire netting)
	kind of technical support to the implementation*	Absence/technician/research project
	payment problems*	yes/no
	distance between footbath and night pen	≤209 m/210–427 m/428–1,188 m/>1,188 m
	technical difficulties	yes/no
	Manager skills	Literate/illiterate, illiterate but helped by a literate person
	treatment problems	yes/no
	absence of water training sessions before treatment	yes/no
**Typology of the farming unit**	cattle breed*	cross-bred zebu, cross-bred exotic, pure exotic, Fulani zebu
	use of a metallic pen*	yes/no
	instruction level*	elementary school, secondary school, traditional
	number of individual facilities*	≤1, 2–3, 3
	type of activities lead by the GLK*	none, without financial management, with financial management
	importance of ticks as a constraint	first constraint, second constraint, third constraint, not cited among the three first constraints
	farmland ownership	yes/no
	number of collective facilities	0, 1, >1
	ratio of resident cattle	≤0.1/0.1–1/1
	presence of herds during May	yes/no
**Farmer perceptions**	efficiency of footbath against ticks*	absence, good, partial
	ease of use of the footbath*	constraining, easy

The ten most contributive variables to the overall variance are marked with *.

Multiple correspondence analysis (MCA) and hierarchic ascending classification (HAC) were used to explore these two sets of variables. MCA allowed highlighting correlations between variables, associations between variables and statistical units (farmers). HAC was used to build clusters of similar farms according to the variables [28]. MCA is an extension of correspondence analysis allowing analyzing the pattern of relationships of several categorical variables. As such, it can also be seen as a generalization of principal component analysis (PCA), when the variables to be analyzed are categorical instead of quantitative. Quantitative variables have first been coded into categories on the basis of quartiles of their empirical distribution. All the variables were then split into categories, and a principal component analysis was used to compute projection axes (factorial axes), constrained to be orthogonal in pairs, the first axis explaining the highest possible variance, and subsequent axes having the same constraint on the residual variance. Only the factorial axes explaining a large proportion of the overall variance were selected to describe the data. Initial variables and statistical units (farms) were then projected into this new set of axes. HAC was used to identify clusters of farms sharing similar factorial coordinates. Ward's criterion was used to aggregate the farms into clusters, thus minimizing within-cluster variance, and maximizing between-cluster variance. A dendrogram of the resulting hierarchy was used to discriminate farms into classes. For this purpose an empirical trade-off was found between the amount of variance explained by the partition, and a minimum number of classes, according to the parsimony principle. The ten most contributive variables to the overall variance were used to describe the groups of farmers. In each cluster, category frequencies for each variable were compared to their frequency in the whole sample using test values [28], [29].

To describe the adoption of footbaths, 7 quantitative variables were used as indicators:

- the individual use of the footbath, corresponding to the number of rainy seasons (RS) during which the farmer used the footbath,
- the ratio between the length of individual use and the number of years of existence of the footbath,
- the ratio between the number of user herds and potential ones,
- the ratio between the number of treated cattle and potential ones,
- the frequency of treatment during the month of June of the last year of use,
- the number of months of use per year,
- the total number of cattle crossing the footbath (number of heads * number of treatments) during the last year of use.

These quantitative variables were submitted to a PCA. The clusters of farmers characterized by their practices were projected on the first plane of the PCA to compare their adoption intensity. The adoption indicators were then compared between clusters previously identified from their breeding practices using a Kruskal-Wallis rank sum test [30]. When the overall effect was significant, bivariate comparisons were done using a multiple comparisons Steel test [31].

All the statistical analyses were achieved using the R software package [32]. MCA and PCA were done with the ade4 package of R functions [33].

### Ethics statement

All farmers provided informed consent before filling the forms. The consents were oral to ensure equal treatment of the subjects, since a large part of the farmers were illiterate (72%). The use of oral consent was approved by the ethics committee of CIRDES and was documented as the first question of all the forms used in this study, after presentation of its goals.

## Results

### Contribution of the descriptive variables to the overall variance

**Figure 2:**
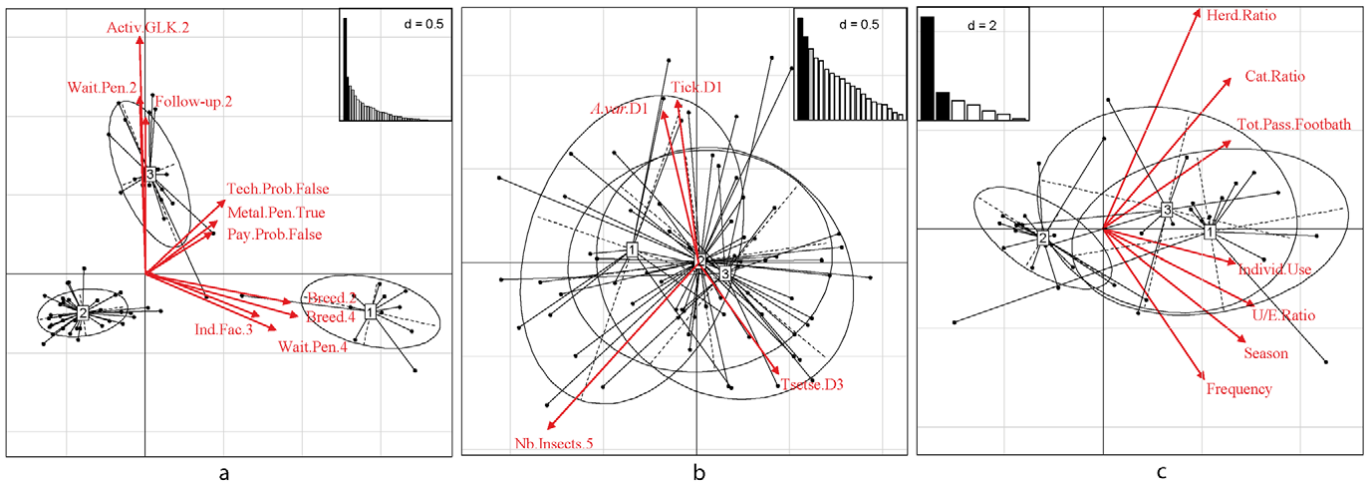
Characterization of the three clusters of farmers using multivariate analyses. From the left to the right, projections of the three groups of farmers discriminated by the ascendant hierarchical classification on the first planes of (a) the MCA applied to the breeding practices and perceptions of the farmers, (b) the MCA applied to the knowledge variables, and (c) the PCA applied to the adoption indicators respectively. The most important modalities are represented by red arrows: Axtiv.GLK.2, absence of financial activity in the GLK, Wait.Pen.2, use of an intermediate waiting pen; Follow.up.2, technical support provided by a research project; Tech.Probs.Fales, absence of technical difficulties; Metal.Pen.True, use of a metallic pen; Pay.Prob.False, absence of payment problems; Breed.2, cross-bred with European breeds; Breed.4, pure European breeds; Ind.Fac.3, more than three individual facilities; Wait.Pen.4, stalling; Tick.D1, ticks not identified as dangerous; A.var.D1, *A. variegatum* not differentiated from other tick species; Tsetse.D3, tsetse diagnosed and vectorial role known; Nb.Insects.5, number of insect categories considered as dangerous; Herd.Ratio, ratio of the number of treated herds on those having access to the footbath; Cat.Ratio: ratio of the number of treated cattle on those having access to the footbath; Tot.Pass.Footbath, total number of cattle passed through the footbath during one rainy season; Individ.Use, number oy years of individual use; U/E.Ratio, ration of the time (years) of individual use on the time of existence of the footbath; Season, duration (months) of the treatment period during the last year of use; Frequency, frequency of use during the last rainy season (June).

**Figure 3:**
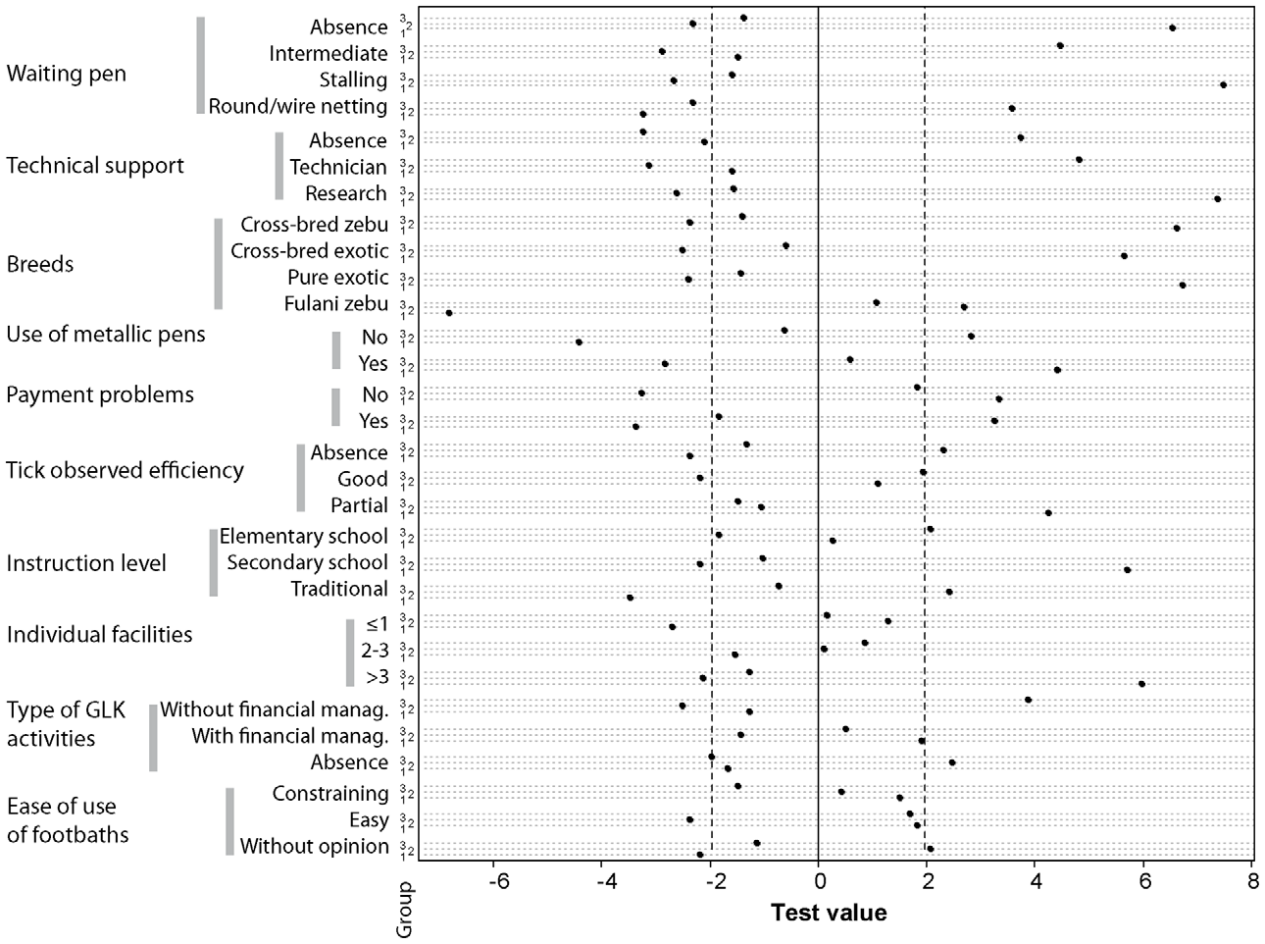
Test values per modality of the main variables describing farmers groups. The central black line corresponds to the median frequency of the modality in the population and the dotted lines to its 95% confidence interval.

Among the variables describing the practices and perceptions of the farmers, the ten most contributive to the overall variance were the type of waiting pen, the technical support, the cattle breed, the use of a metallic pen, the payment problems, the observed efficiency against ticks, the type of instruction of the farmer, the number of individual facilities, the kind of activities carried on by the GLK and the easiness of use of the method (individual perception). Their modalities were well discriminated by the first plane of the MCA (Fig. 2), and their frequencies were different between groups (Fig. 3), in particular for the type of waiting pen, the technical support, and the cattle breeds.

### Correlations between descriptive variables

The use of a stalling as a waiting pen, characterizing the modern farmers, was the most contributive category to the first axis of the MCA. It was highly correlated with a high level of instruction (high school and more), to the use of improved breeds (pure European breeds and cross-bred with European breeds), to a low distance between the footbath and the night pen, as well as a technical support by a technician, the absence of collective facilities, numerous individual facilities (more than 3 categories), ticks as an important constraint (third constraint to cattle breeding in general) and a partial observed effect of the footbath against ticks (p<0.05, Fig. 2). The use of a metallic pen (waiting pen or stalling) was associated to an absence of difficulty for the cattle to cross the footbath and to an absence of payment problems (p<0.05, Fig. 3), as well as a positive assessment of the easiness of use of the method.

The absence of financial activities in the GLK was the most important modality on the second axis. It was correlated with the use of intermediate waiting pens (funnel shaped with wire netting), and with a technical support by a research project, as well as with a large distance between the footbath and the night pen (3^rd^ quartile, from 787 to 1,188 m) (p<0.05, Fig. 2).

### Description of the breeding systems in the farmers groups

**Figure 4:**
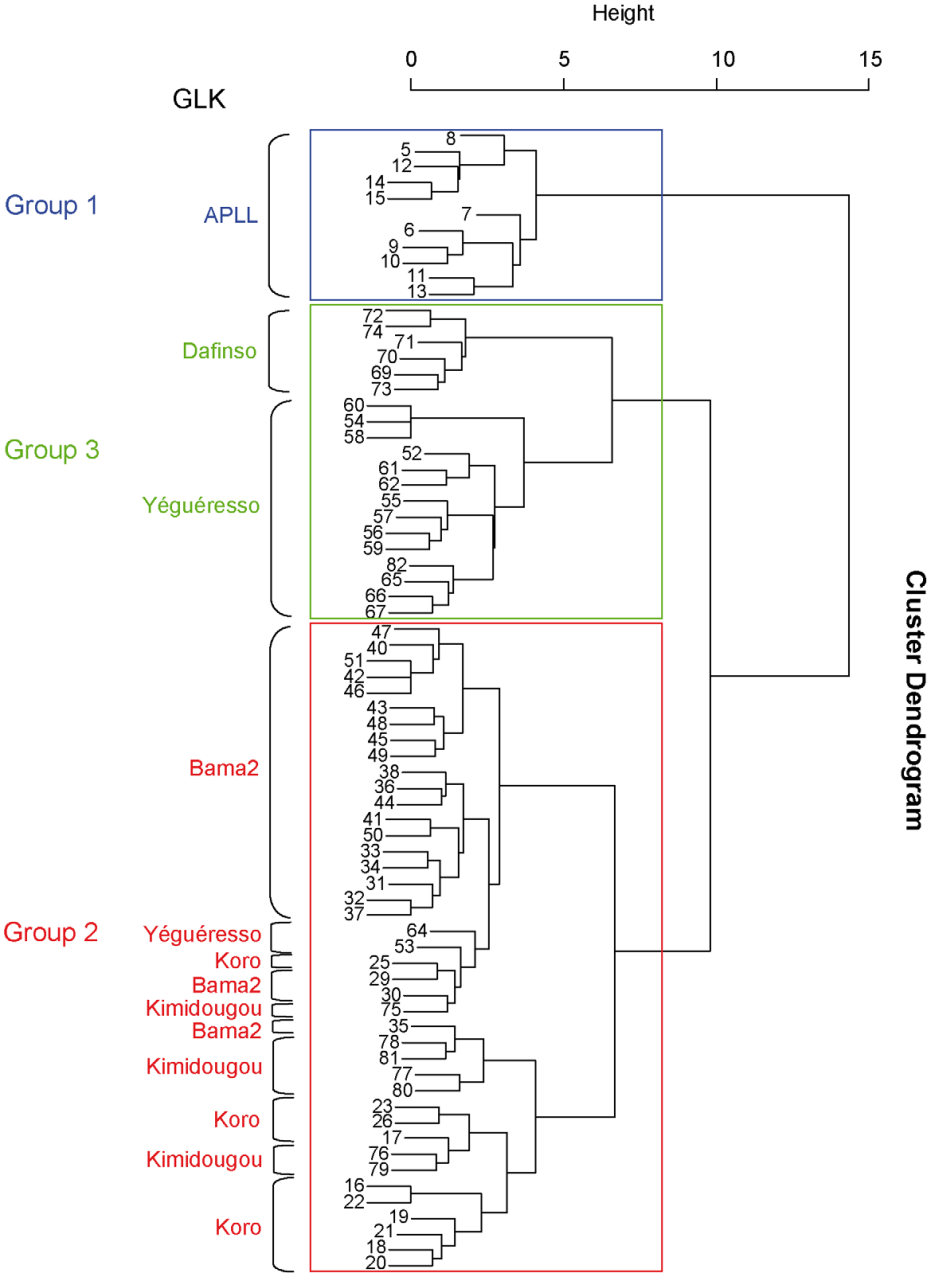
Cluster dendrogram of the farmers. The farmers were discriminated in three groups using a hierarchical cluster analysis after a MCA applied to of their practices and perceptions (Ward's method).

The three groups were well discriminated by the first factorial plane of the MCA (Fig. 2). Group 1 was discriminated from the two others by the first axis; the second axis discriminating group 3 from the two others. Projections of farmers belonging to a GLK were generally close to each other on this factorial plane, since some descriptive variables were measured at the scale of the GLK. However, such closeness was not systematic. For example, two farmers of the Yegueresso GLK belonged to a group different from other members (Fig. 4).

The first group included 11 farms corresponding to the ten Ouagadougou farmers (one of them owning two farms). The second group included all the surveyed farmers (*n* = 41) of 3 GLK (Koro, Bama 2 and Kimidougou) plus two Yegueresso farmers. The last group included most Yegueresso farmers, and those of Dafinso (Fig. 4).

Group 1 (Ouagadougou farmers) was associated with item modalities corresponding to modern cattle breeding systems, i.e. stalling as a waiting pen (91%), and sedentary cattle (grazing area close to the stalling, during the rainy season only). They mostly (82%) benefited from a technical follow up by one of the farmers belonging to the same GLK (who was also a consultant in livestock farming). Local Fulani zebus were found in a single farm (9%), whereas the most frequent cattle type was zebu cross-bred with European breeds (45%). Pure European breeds, and some exotic zebu breeds (Goudhali, Azawak, etc.), were also observed. All the farmers belonging to group 1 used a metallic pen (stalling or vaccination corridor). Payment problems did not occur since the footbaths were used individually. A large majority (73%) of the farmers had a high level of instruction (at least secondary school level). The farmers owned at least 3 categories of individual facilities. On the other hand, collective facilities were scarce (18%). The GLKs lead activities involving financial management. A majority of the farmers (55%) observed a good efficiency of the footbath, whereas one third reported a partial efficiency, and 9% did not observe any impact. A large majority of the farmers (73%) found the footbath convenient and easy to apply. In all the farms, the footbath-night pen distance was <209 m, conversely to groups 2 and 3. Indeed, the stalling was used as a waiting pen, and footbath was built at its exit. Only one third (27%) experienced technical difficulties. This group was not subjected to any nagana risk and could thus not appreciate the impact of footbaths on tsetse.

Groups 2 and 3 were traditional farmers of the peri-urban area of Bobo-Dioulasso belonging to the UEPL cooperative (Houët dairy farmers union).

Group 2 was the largest cluster of farmers (*n* = 41). All the footbaths had a round waiting pen with wire netting. No technical follow up of the service implementation was provided (after the initial technical training of the elected GLK members achieved at CIRDES). Herds were made of local Fulani zebu (with some cross-breeding with trypanotolerant cattle). A single farmer used a metallic pen (vaccination pen). Up to 98% of the farmers experienced payment problems. Farmers' education was mostly traditional (93%). Most group-2 farmers (70%) owned very few individual facilities (1 at the most), but collective facilities were frequent (80%). Most of them (78%) were unable to judge the ease of use of the footbath because they hardly used it, if ever. Many of the GLK had no other activity than representation (54%). Others had activities involving financial management (46%). 83% of the farmers did not observe any effect of the treatment on ticks, in relation with the low footbath usage in this group. Only 13% of the group-2 farmers reported a good efficiency against ticks, whereas 5% observed a partial effect. Few night pens were located close to the footbaths (10%), whereas 34% were located 209 to 427 m from it, and 44% even further (>1,188 m). All the farmers reported technical difficulties. Only 5% this group observed a reduction of nagana risk thanks to the use of footbaths.

Group 3 included 20 farmers owning mainly intermediate waiting pens (75%) whereas only 25% were round pens with wire netting. This group mostly benefited from a follow-up survey implemented by the research team (95%). Indeed, the two footbaths implemented by the CIRDES belonged to the Yegueresso GLK which was well represented in this group. Moreover, the CIRDES used the Dafinso GLK to measure the efficiency of the method against tsetse during one year. Lack of technical follow up after footbath installation only concerned 5% of the group. The main breed was the local Fulani zebu (95%), whereas very few cross-bred cattle with European breeds were observed. One third of the farmers used a metallic pen, and one third experienced payment problems. They were mostly traditionally educated (60%), but 35% went up to the elementary school level. Like group-2 farmers, they owned very few individual facilities (1 at the most for 60% of them), but 90% of them used collective facilities. Sixty-five p. cent of the farmers found the footbath easy to use. The majority (70%) of them belonged to a GLK providing financial management. Most group-3 farmers have mostly (65%) observed a good efficiency of the footbath against ticks whereas 35% did not observe any effect of the treatment. Few night pens (15%) were close to the footbaths. The majority of these night pens (55%) were located between 787 and 1188 m from the footbath. Technical difficulties were very frequent (70%). Only 15% of this group observed a reduction of nagana risk thanks to the use of footbaths, and 5% considered that it stopped nagana transmission.

### Epidemiological knowledge in the three groups

The three groups showed a similar knowledge of the epidemiological system, as demonstrated by their important overlapping on the first plane of the CMA applied to the knowledge variables (Fig. 2b). Groups 2 and 3 harboured a nearly complete overlapping which showed that the two groups of traditional herders mostly shared the same knowledge whereas group 1 did not overlap completely.

The v-tests applied to 6 representative variables mainly confirmed this result (see figure S1). However, some minor trends were observed: group 3 that benefited from technical support by the research team better knew the pathogenic impact but were not able to recognize tsetse species more than group 2. Diagnostic mistakes for tsetse were however the most frequent in group-1 farmers (Ouagadougou) who live in an area where tsetse flies were absent.

### Adoption intensity in the 3 farmer groups

Projection of farmer groups on the first factorial plane of the PCA applied to the adoption indicators (Fig. 2c) showed that group-2 farmers were well discriminated with respect from the other groups. Group-1 and -2 famers overlapped mostly on the first axis of the plane (56% of the global inertia). Group-2 farmers did not adopt the footbath, whereas the two others showed better and similar adoption levels, though they represented different cattle farming and footbath management systems: individual management in group-1 farmers, versus collective management in group-3 farmers.

**Figure 5:**
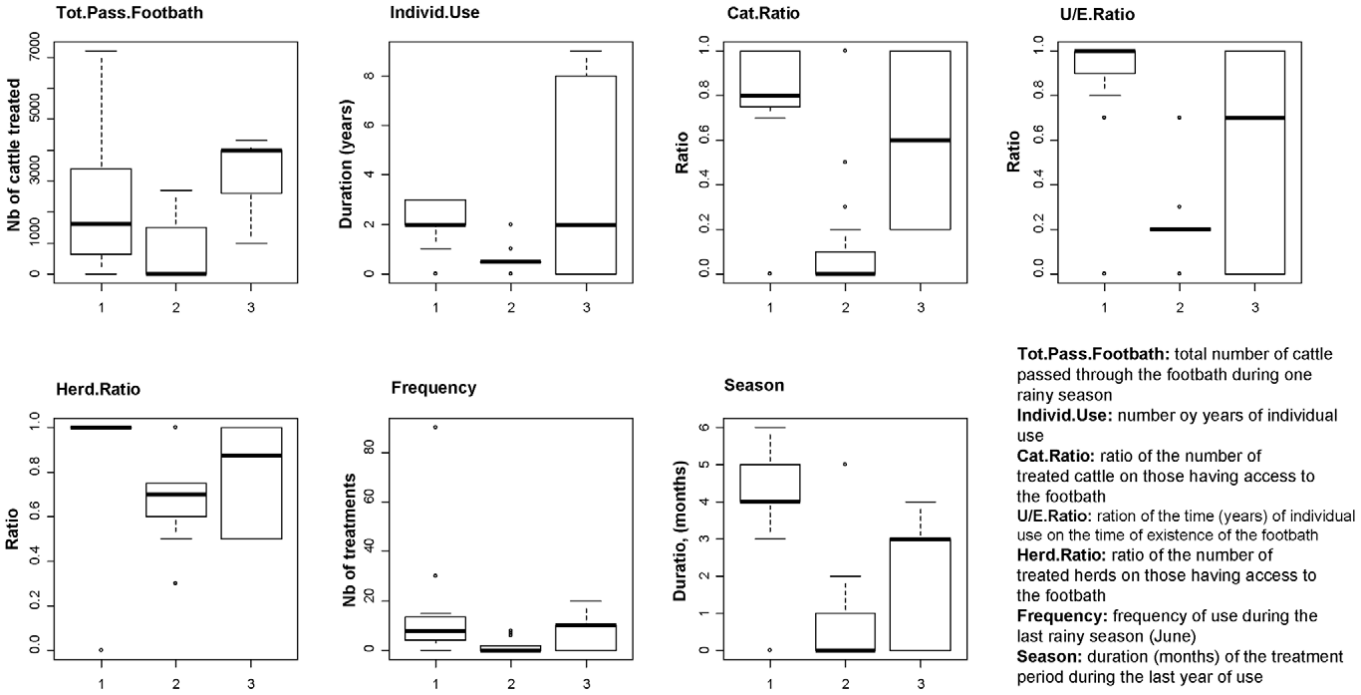
Distributions of the adoption indicators within farmers groups. Boxes and whisker plots presenting the simplified distributions (quartiles, median, 95% intervals) of the adoption indicators in the 3 farmers groups (x axis).

All the adoption indicators were defined so that they should increase with the intensity of adoption. Adoption patterns were different according to each indicator (Fig. 5), confirming that they represented different features of adoption. This was confirmed by the absence of correlation between these variables (p>0.05; Fig. 3).

In group-2 farmers, values taken by the indicators were always low, and lower than in the two others farmer groups (p<0.05). Medians were equal to zero for (i) the total number of cattle treated with the footbath, (ii) the ratio between treated cattle and potential users, (iii) the monthly frequency of treatment, and (iv) the duration of annual use. The median of the duration of individual use was only 0.5 rainy season (RS), corresponding to occasional tests during the first year of use (i.e. less than one complete season). The median of the total number of treatments was 0.5 but the third quartile at 1,500 revealed a strong variability. The ratio between the duration of use and the duration of existence of the footbath was also low in this group (median at 0.2 against 0.7 (p = 0.03) and 1 (p<0.001) in groups 3 and 1 respectively). The adoption rate was thus very low in this group.

The median of the total number of treatments per footbath during the last rainy season of use was highest in group 3 (4,000) but the highest maximum (>7,000) was observed in group 1, whose variability was higher. The difference between the mean values (3,494 and 2,377 treatments in groups 3 and 1 respectively) was not significant (*p* = 0.07). The number of cattle having potential access to the footbath was however higher in group-3 farmers (collective use). Duration medians of individual use (number of RS) were identical in these groups (two years, *p* = 0.83) but the variability was much higher in group-3 farmers, with the highest maximum (nine years) corresponding to the first footbaths built by the research team. The ratio between the duration of individual use and the existence of the footbath was highest in group-1 farmers (median of 1, first quartile close to 0.90 and mean value of 0.86). Group-3 farmers harbored lower values (median of 0.70, mean value of 0.53, p = 0.04), intermediate between the two others groups. Concerning the ratio between the treated cattle and the potential users, mean values were similar between the groups (0.80 and 0.60 in group-1 and group-3 farmers respectively, *p* = 0.45), but more variable in group-3 farmers. At the herd level, this ratio was 1 for all farmers but one in group 1, which reflected the individual-use feature. In group-3 farmers, median was also high (0.90) and not significantly different at the herd level (*p* = 0.067), although more variable. Monthly frequencies of treatments were also similar in group-1 and -3 farmers, with medians corresponding to the technical recommendations (10 and 12 respectively). However, we observed two ectopic values (30 and 90) in group-1 farmers, corresponding to three treatments per day! Mean frequencies were similar between farmer groups (17 and 18 for group-1 and -3 respectively, p = 0.99). Finally, the duration of use during each rainy season was lower in group-3 than group-1 farmers (mean value of 2.2 and 3 months respectively, p = 0.002), corresponding to transhumant practices.

### Geographical location of the farmers

In Bobo-Dioulasso, the ratio between the duration of individual use and the existence of each footbath (U/E) was projected on the map (Fig. 1). The herders of the Yegueresso and Dafinso GLKs appeared well discriminated in space and regarding this ratio. This reflected their collaboration with CIRDES: the two first experimental footbaths were built in Borodougou and Tondogosso, belonging to the Yegueresso GLK, where a 4-year technical follow up was implemented to assess their efficiency against *A. variegatum*. In 2007, the highest number of new footbaths (4) was built in this GLK. Similarly, Dafinso was the place where the efficiency of footbaths against tsetse was demonstrated, during the rainy season 2007. In addition, group-3 farmers were generally located closer to Bobo-Dioulasso than the others, and along the main roads.

## Discussion

### Practices, knowledge and adoption

Rural knowledge and cultural conceptions are considered to be crucial to explain farmer practices and their evolution. In our study however, we did not observe significant differences concerning the epidemiological knowledge of the 3 groups. Practical knowledge (e.g., accurate diagnostic of insect species) was similar in modern and traditional farmers, even if concept formulations were different. However, instruction level was among the ten more discriminating variables between farmer groups. Indeed, the highest instruction level (provided by the school) corresponded to farmers being more sensitive to scientific concepts of modern cattle farming. This variable was also partly linked to the cattle farming system because farmers with a second professional activity could more easily fund innovations and take more economic risks for their cattle farming activity. Moreover, people living far from urban centers and communication ways belonged to different socio-technical networks and more traditional social systems in which school frequentation was lower, for instance. Indeed, if a research center has to choose between two equivalent study areas, the closest and most accessible will often be selected. Therefore, elected people and the members living close to the main town had more socio-technical exchanges with various partners. As evidence, four footbaths were implemented in the Yegueresso GLK where the first footbaths were built, and the UEPL president used to live.

The two perception items belonging to the ten most important variables were (i) the farmers' perception of the footbath efficiency against ticks (generally considered as their first motivation to use insecticide treatment of cattle [34]), and (ii) the farmers' perception of the easiness of use of the method. Unfortunately, these perception features are not known in advance, and cannot be used to select potential beneficiaries having greater chances to adopt the method in the future. It is clear from this study that the impact of footbath against tsetse and trypanosomoses was not their first motivation for adoption of the technique, since the control group, located in an area without tsetse, harbored a good adoption rate. Even in the tsetse area, the treatments with footbath were limited to the rainy season, i.e. the period of infestation of cattle by the tick *A. variegatum*. It must be noted however, that treating cattle during the rainy season is enough to prevent trypanosomoses throughout the year in this area [20]. The high rates of dissatisfaction with the footbath against ticks are probably related to two main causes. First, the footbath treatment is not designed to kill the ticks that are already attached to their predilection sites but to prevent new infestations, which gives a negative perception of its efficiency. Second, group 2 did not use the footbaths enough to appreciate its efficiency (median frequency of use of 0) whereas half of the farmers of groups 1 and 3 did not apply the recommended treatment frequencies (medians corresponding to the recommendations) which is upon the rates of dissatisfaction. The efficiency of this technique against *A. variegatum* was confirmed when the appropriate treatment frequency is applied [17] and we did not observe any resistance of ticks against pyrethroids in our study area despite several resistance trials conducted at CIRDES (Adakal, pers. com.).

The practical modalities of footbath implementation, described by the type of waiting pen, the technical support, the distance between footbaths and night pens, and the financial and technical difficulties, appeared preponderant to explain footbath adoption rate in this study. These criteria characterized the difficulty (or conversely the ease) of use of the method [35]. Decreasing the technical constraints related to footbath treatment has always been of a concern since its invention, for example by recommending the respect of a low distance between footbaths and night pens [17]. The technical support provided to the traditional farmers appeared insufficient for those not involved in research projects, i.e., most of them. In addition to a technical support, a regressive financial support was also brought to those involved in these projects, decreasing the risk undertaken by the herders. Another important element that did not appear in the study is the fact that the two footbaths implemented within research projects were each managed by a single family. Members of the dairy-farmer union (APLL) not only owned more individual facilities, but also benefited from a better support by a technician. Their footbaths were all managed individually, thus eliminating issues related to collective management practices.

The collective management of such innovation may also raise difficulties related to its public-good nature. Indeed, in the context of cattle farming in Burkina Faso [15], as well as in most Sub-Saharan African countries [34], farmers show a clear preference for individual control methods and the borrowing of individual facilities. The change from an individual to a collective mode of management represented an important social change, and a difficult step to overcome. In this situation, restricted application of insecticides using hand spraying might be a better alternative, even if more time consuming [4].

The nature of the waiting pens was very different between the three groups and had an important impact. The most favorable layout was the use of the stalling as a waiting pen, as observed in group-1 famers. Conversely, all group-2 farmers owned round waiting pens surrounded with wire. They experienced a lot of technical difficulties, especially to make the cattle walk through the footbath, which sometimes was even impossible because cattle did not perceive well the way to follow. The traditional night pens made of branches appeared more suitable, because they were more familiar to cattle.

The analysis of the financial problems met for footbath management was difficult in this study because they were either a cause or consequence of a lack of adoption. Actually, the financial management of a collective good was a problem. It was also an aspect of the implementation modalities for which farmers had to be trained. When farmers refused to pay the service because they didn't want to adopt the method for some reason, payment difficulties then became an indicator of adoption. The relative importance of these two phenomena was difficult to assess in this study.

The cattle farming system (described by the cattle breeds, the use of a metallic pen, the number of individual facilities and the type of activities lead by the GLK) appeared very important here and could have been used to predict the adoption level. The kind of activities lead by the GLK indicated its management type, the level of strengthening of the farmers' capacities and the dynamism of the production system. While the APPL was able to find internal resources to provide a technical follow up for footbaths implementation, it was not possible for the UEPL. In the latter case, without an external follow up (as provided by research projects), difficulties were sometimes impossible to overcome. Economical aspects could not explain the lack of adoption of the method because in all the villages where adoption failed, the number of footbath treatments was <4,000, i.e., below the insecticide stock provided by the project. But, as stated by Alary (2006) in another context, “the structural factors and the economic logic cannot explain all the adoption process. The social or even moral supports, provided by the development agents and the researchers, have played their role too.” In some GLKs, sociological blocking has occurred, corresponding to situations outlined by Alary (2006): “the mistrust between producers prevents intra-community changes in the absence of interference of external agents.” That is why communication and debates within the farmer groups and socio-technical network are very important to explain the advantages of the proposed innovation process [36].

Concerning cattle breeds, one might argue that this item was a confounding factor for better education and management processes associated with modern farmers. However, one of the group-3 farmers stated that cross-bred cattle (with European breeds) sharing the same night pen than their Fulani zebus, accepted to walk through the footbath more easily than the latter. Other farmers confirmed that Fulani zebus experienced more difficulties than European or cross-bred cattle to use footbaths. This was not surprising because European breeds have been selected on behavioral features, including tameness [37], while Fulani zebus have probably been selected through centuries by African pastoralists on their nervous behavior and their capacity to be easy to handle only by their herder/owner [38]. On the same ground, the item “regular use of a metallic pen” (stalling or vaccination pen) was noteworthy because of its predicting value for adoption intensity. A learning behavior of cattle was probably involved there, also explaining why the waiting pens made of branches were more appropriate in traditional farming systems, because they looked much like traditional night pens.

### Adoption and risk appraisal by the farmers

Why are smallholders from developing countries often reluctant to technologic innovations [39]? Five criteria have been proposed to assess the adoptability of innovations [35], [40]: (i) the relative advantage brought by the innovation in comparison to the initial situation, (ii) its compatibility with the current system, (iii) its complexity, (iv) its “triability” in the farmers' context (possibility to test the technique), and (v) its “observability” (possibility to observe the technique used by other farmers). Indeed, the advantage/risk ratio appraisal should be obviously beneficial for a good adoption by farmers.

The relative advantage of the footbath in comparison to other vector control methods has been assessed in experimental and field conditions: it's an efficient, cheaper and less time-consuming method [17], [19]. The items “technical difficulties”, “difficulties of treatment”, “efficiency against ticks” and “easiness of use” have contributed to the assessment of this criterion which was much different across the farmer groups, because it depended on the way the service was implemented. Footbath “triability” was low on average, because few farmers were close enough to a footbath to test it. Even its “observability” was moderate in the GLK where footbaths were more observable and triable (Yegueresso), the adoption rate was higher. Finally, the compatibility with the current system, and the complexity of the method (2^nd^ and 3^rd^ criteria), were assessed together by items describing either the production system or the socio-technical parameters (such as the kind of activities conducted by the GLK). It was not possible to give an accurate assessment of each footbath-specific criterion because their distribution was very different across farmer groups. For example, treatment difficulties were a strong constraint for the traditional farmers, but not for the modern farmers.

The good adoption level in the modern farmers of Ouagadougou was not surprising because the farmers were already engaged in an intensification strategy: they already invested in modern facilities (metallic pen, vaccination tunnel, etc), sometimes expensive if the potential technical/economical benefit were important. The implementation of a footbath did not represent a large financial, technical, or social risk. The individual use of these facilities had no social impact in this group. On the other hand, the footbath represented a more important risk for the traditional farmers. Indeed, the farmers mentioned that the cattle could not be treated during their transhumance, when ticks are the most abundant. Therefore, they may have underestimated the economic advantage of the footbath because they focused mainly on its impact on ticks rather than tsetse (which are present throughout the year). While the farmers did not invest much money in the footbaths, they had to spend time to get the cattle used to cross the footbath, and make efforts (training) to understand and apply the technical requirements (dosage of the insecticide, filling of the forms, etc.).

Moreover, the collective use of the footbaths had a social impact. The footbath managers got a strategic function since they were in charge of the footbath maintenance, and they had to attend all the footbath treatments, and had to record the exact number of treated cattle for each farmer, and to calculate the amounts to be paid by each of them. These managers had to be available (almost every day, which was a very limiting constraint), and to know how to read and write relevant data, and to be able to understand the management documents (abacus, treatment forms)… Therefore, young educated people were often selected as managers. These “children” were also selected because they were obliged to their seniors who considered that they should not be paid for this service nor manage the financial aspect of the activity. Moreover, their new functions conferred them a new strategic position which changed the former social relationships. Indeed, this competition with the traditional authorities can sometimes lead to conflicts, particularly concerning the management of natural resources [39]. When traditional social systems are subjected to tough conditions, and their economical survival depends on hazards (climate, diseases, etc.), their resilience relies on a strong solidarity, and on conservative attitudes, aiming at keeping the economic sustainability of families [39]. Any change of the social system is thus considered as an important stress possibly impacting risk perception related to innovations.

### Recommendations to tsetse control projects

Finally, it must be acknowledged that in other areas, the positive impact of the method on human health might also favor its adoption, a phenomenon that we could not study here. Actually, restricted application of insecticides combined with trypanocide treatment of cattle, might provide locally effective control of Rhodesian sleeping sickness (*T. brucei rhodesiense*) and diminish the trypanosome reservoir in cattle hosts during inter-epidemic periods [9]. In the case of the Gambian sleeping sickness (*T. brucei gambiense*), footbaths might also allow a reduction of disease transmission through a reduction of tsetse density: recently in Chad, in the area of the active focus of Mandoul [41], footbath treatment of cattle herds thus allowed to reduce by 95% the density of *G. f. fuscipes*, the main vector of sleeping sickness (Bouyer, pers. com.). Moreover, it has been suggested that it could help in controlling Malaria [42], within the framework of the One World, One Health' concept (http://www.oneworldonehealth.org/). However, underlying concepts are much more difficult to explain in this case: insecticide treatment to break the trypanosome transmission cycle in cattle and thus suppress the reservoir for human infection in the case of Rhodesian sleeping sickness, and to reduce the relative density of tsetse to humans in the case of Gambian sleeping sickness. Therefore, it would necessitate careful training of stakeholders, as well as relevant information for farmers and community medical health workers.

However, the adoption factors identified in this study still allow for provision of the following recommendations to future tsetse control projects willing to include a farmer-based component:

- favor tsetse control strategies that protect a private good and are effective against ticks, i.e. insecticide treatment of cattle, since the control of ticks is the main driver for farmers' active use of the method [34]; in Burkina Faso, even farmers identifying tsetse and trypanosmoses as their main constraint did not perceive the benefit of the method;
- allow the observability and triability of the method throughout the target area, before the start of the tsetse control campaign;
- strengthen the GLK through the organization of activities including a financial aspect and allowing them to provide technical services to their members.

In the particular case of restricted application of insecticides, the following advices can be laid:

- diagnose the farming systems and more importantly the socio-technical network of the targeted farmers; if modern farmers belonging to dynamic GLK associations with financial activities are to be involved, the use of footbaths is advisable, with a short-term technical follow-up; if traditional farmers belonging to dynamic GLK associations with financial activities are to be involved, the use of a footbath is advisable, but a long term technical follow-up (at least for 3 years) is necessary; if traditional farmers belonging to GLK with a representative role only (or not belonging to any GLK) are to be involved, collective control is not the best option and individual treatments using hand spraying is probably better [4] (but in this case, the efficiency of the method is questioned since the rate of cattle treated in a given area might be insufficient [21], particularly in the presence of a tsetse re-invasion pressure [34]).
- an individual footbath management is more suitable to ensure a successful adoption, but a collective management is possible, at least at the family level;
- the waiting pen should be fully considered as a part of the innovation, and best built at the exit of the stalling for individual footbaths or funnel-shaped with materials similar to the night pen for the collective ones, to reduce the technical difficulties of treatment; the quality of the waiting pen is all the more important for local breeds.

## Supporting Information

Figure S1 **Test values per modality of the variables describing farmers'knowledge of the epidemiological system.** The central black line corresponds to the median frequency of the modality in the population and the dotted lines to its 95% confidence interval.
(TIF)
