# Supplementary figures and images for: Restricted Application of Insecticides: A Promising Tsetse Control Technique, but What Do the Farmers Think of It?
Source: PLoS Negl Trop Dis. 2011 Aug 9;5(8):e1276. doi: 10.1371/journal.pntd.0001276 (PMC3153426; doi:10.1371/journal.pntd.0001276)

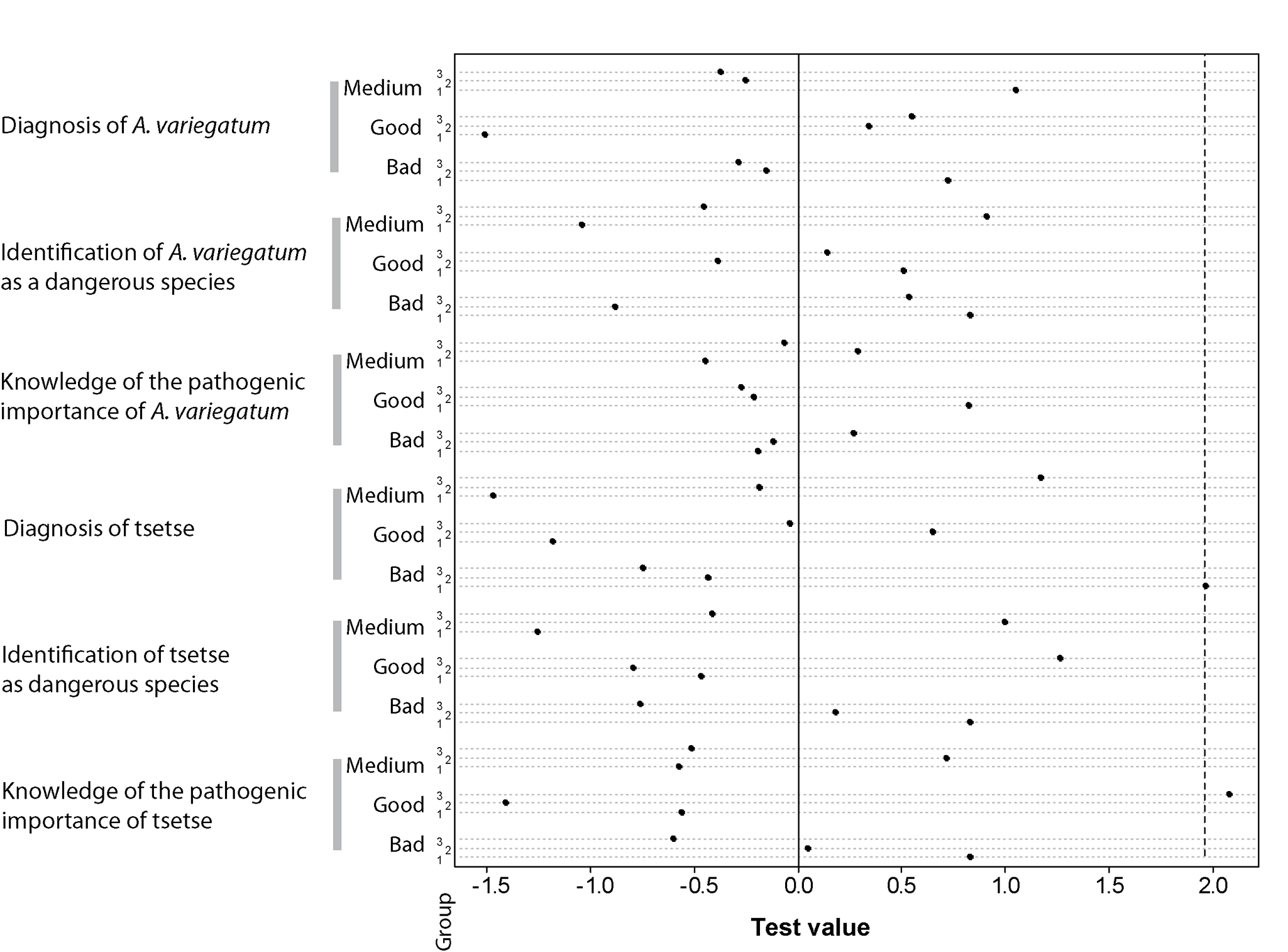

Supplement: Figure S1 — Test values per modality of the variables describing farmers'knowledge of the epidemiological system. The central black line corresponds to the median frequency of the modality in the population and the dotted lines to its 95% confidence interval. (TIF) [file pntd.0001276.s001.tif]
